# The Association Between Connectedness and Grit Among Thai In-school Adolescents in Urban Chiang Mai, Thailand

**DOI:** 10.3389/fpsyg.2022.809508

**Published:** 2022-03-28

**Authors:** Arunrat Tangmunkongvorakul, Matthew Kelly, Kulvadee Thongpibul, Patou Masika Musumari, Kriengkrai Srithanaviboonchai, Cathy Banwell

**Affiliations:** ^1^Research Institute for Health Sciences, Chiang Mai University, Chiang Mai, Thailand; ^2^Department of Global Health, Research School of Population Health, Australian National University, Canberra, ACT, Australia; ^3^Interdisciplinary Unit for Global Health, Center for the Promotion of Interdisciplinary Education and Research, Kyoto University, Kyoto, Japan; ^4^Faculty of Medicine, Chiang Mai University, Chiang Mai, Thailand; ^5^National Centre for Epidemiology and Population Health, Research School of Population Health, Australian National University, Canberra, ACT, Australia

**Keywords:** Grit, students, connectedness, parental involvement, Thailand

## Abstract

**Aim:**

To investigate the associations between Grit, connectedness, and parental involvement in Thai adolescents. Grit, perseverance, and passion for long-term goals are predictors of academic success and health. There is a small but developing knowledge of the predictors of Grit in Asia, especially Thailand. This paper investigates the proposition that connectedness and parental involvement are positively associated with Grit.

**Method:**

A total of 2,839 lower secondary (grade 8), higher secondary (grade 11), and vocational (year 12) students from 21 schools in Chiang Mai, Thailand participated in a survey that measured Grit using the Short Grit Scale. Bivariate analysis was conducted using the *t*-test, ANOVA, or Kruskal–Wallis H test as appropriate. Multiple ordinary least squares linear regression analysis was performed to determine factors associated with Grit.

**Results:**

Satisfactory relationships with teachers (*p* = 0.01), parental support (*p* = 0.03), interest in school (*p* = 0.01), having been asked by parents to do homework (*β* = −0.69; *p* = 0.012), and having been told by parents that they had done something bad (*β* = −1.09; *p* = 0.02) associated with Grit. These findings can aid in design of tailored interventions to improve Grit in Thai adolescents.

## Introduction

Researchers have long been interested in identifying factors that contribute to academic attainment and a healthy successful life. One non-cognitive factor that has rapidly captured the attention and curiosity of educators, researchers, policymakers, and the public as a strong predictor of academic, health, and life success is Grit (Duckworth et al., 2007; Duckworth and Quinn, 2009). Grit has been examined for its contribution to academic and work-related success in Western countries. Thus far, it has not been extensively investigated in developing Asian countries with lower educational resources. Individual student characteristics, such as effort, make a significant but slighter smaller contribution than socio-economic background to student performance (Asadullah et al., 2021) while high performing South-East Asian education systems such as Vietnam appear to benefit from favorable individual cultural capital characteristics such as Grit, discipline, or perseverance. This study focusses on Thailand which does not perform as well on Grit or on educational performance (Asadullah et al., 2020), as some other Asian countries. Given Grit’s potential role in contributing to academic success and the need to improve Thailand’s educational performance, it is useful to explore factors, such as connectedness and positive parental involvement, that may contribute to improved Grit.

### Theoretical Background

Grit has been conceived as a personality trait and is defined as “perseverance and passion for long-term goals” and as “working strenuously toward challenges, maintaining effort and interest over years despite failure, adversity, and plateaus in progress” (Duckworth et al., 2007). Grit consists of two components: perseverance of efforts and consistency of interest that in combination make the higher-order construct of Grit. Although Grit has been extensively questioned as a construct (Credé et al., 2017), its use is growing as an important component of successful education.

A growing body of research has investigated how Grit is constructed and whether it can be clearly distinguished from similar concepts. For example, a study of nursing students found that Grit is correlated with resilience because it includes persistent effort, although it is also distinguished by “consistent interest” or ability to “stick” when things get difficult (Meyer et al., 2020). Grit also differs from enthusiasm which appears to be important to educational outcomes but can be feigned (Moe, 2016; Moe et al., 2021). Enthusiasm and passion are similar (Keller et al., 2016), although the component of passion in Grit has been described as “strong enthusiasm” (Sigmundsson et al., 2020).

### Grit as Predictors of Life Success

Grit is increasingly seen as important as it has been found to account for variance in successful academic outcomes over explanations provided by traditional intelligence quotient (IQ) tests. It also predicts health behaviors, and life success in the form of marriage stability and employment retention (Duckworth et al., 2007; Reed et al., 2013; Eskreis-Winkler et al., 2014). Those showing Grit were more likely to succeed in various goals: students were more likely to graduate from high school; soldiers were more likely to complete an Army Special Operations Forces (ARSOF) selection course; sales employees were more likely to keep their jobs; and men were more likely to stay married (Eskreis-Winkler et al., 2014). Higher measured Grit is also related to greater healthcare management skills, higher mental health-related quality of life, and higher physical health-related quality of life in college students (Sharkey et al., 2017). In addition, Grit has been identified as a protective factor against substance use and risk behaviors, suicide risks, late-life cognitive impairment, and negative self-concept in children with reading disorders (Kleiman et al., 2013; Guerrero et al., 2016; Thomas et al., 2016; Credé et al., 2017; Rhodes et al., 2017).

Given that Grit predicts academic, health, and life successes and has been identified as a protective factor against negative consequences in life. Examining factors associating with Grit may be a critical step in identifying possible areas of intervention to enhance Grit. It is important to measure Grit among young people as it is associated with future success over the life course.

### Social Connectedness as a Factor for Grit Development

Although Grit is largely contingent on genetic factors (Rimfeld et al., 2016; Tucker-Drob et al., 2016), like other personality traits, it can be influenced by social and environmental factors (Duckworth et al., 2007; Datu, 2017, 2021; Alan et al., 2019). However, most research on Grit thus far has examined its predictive validity toward academic and life success. There is a dearth of research that has examined Grit as an outcome. Few studies have linked Grit to social connectedness. As defined by Catalano et al., connectedness is the emotional attachment and commitment a child makes to social relationships in the family, peer group, school community, or culture (Catalano et al., 2002). It is recognized that the quality of a child’s bonds to family and other domains is an essential element of positive development into a healthy adult. In relationship with Grit, studies among middle and high school students in the United States have shown that factors such as school engagement, relationships with adults and peers, and school culture were strongly related to Grit (Farrington et al., 2012).

As we have noted much of the current knowledge on Grit derives from research in western countries. One of the few studies conducted in an Asian context argues that in collectivist Asian cultures, Grit is positively associated with connectedness or relatedness. A study of Filipino high school students found that having positive relationships with parents and teachers matters for long-term goals (Datu, 2017). However, this study did not include specific factors related to practical areas of parental involvement. A Chinese study found that some Adverse Childhood Experiences (ACEs) that are sometimes associated with the home environment, may negatively impact Grit (Cheung et al., 2021). It seems that sexual and emotional abuse and neglect from ACEs impacts negatively on relatedness (Cheung) which is related to Grit. A recent study that compared Grit and mindfulness between students in New Zealand and Thailand found no difference in effort between the two groups; however, the New Zealand students endorsed higher levels of perseverance of effort relative to Thai students (Raphiphatthana et al., 2019). Another study among high school students in Thailand found no correlation between Grit and success in computer programming learning (Thanasuan et al., 2017). The study was, however, limited by its small sample size (46 participants). Despite increasing interest in Grit globally, few studies thus far have examined the associations between Grit, connectedness, and family involvement anywhere, including Thailand.

### Conceptual Framework

In this study, we use the theoretical underpinning of Grit developed by Duckworth and others as described above to explore the relationship between social connectedness and Grit in Thai in-school adolescents in Chiang Mai. In particular, we investigate the concept of how social connectedness acts in predicting or developing Grit. We hypothesized that connectedness to social agents (parents, peers, and teachers) has positive correlation to Grit in Thai in-school adolescents in Chiang Mai.

## Materials and Methods

### Study Design, Population, and Setting

This is a cross-sectional, quantitative study conducted in urban Chiang Mai between November 2016 and February 2017 designed to investigate associations with Grit and factors that may predict it. For the education system in Thailand, school levels are divided into primary schools (grades 1–6), lower secondary schools (grades 7–9), higher secondary schools (grades 10–12), and vocational schools (years 1–3, which equivalent to grades 10–12 in higher secondary schools). In urban area Chiang Mai City, it has 21 secondary and vocational schools in total, including 15 secondary schools, which having both lower and higher secondary school levels (five public and 10 private), and six vocational schools (two public and four private). Participants were recruited from all 21 schools in urban Chiang Mai. They were recruited from among lower secondary students (grade 8), higher secondary students (grade 11), and vocational school students (year 2).

The recruitment was a two-step process. Cluster random sampling was conducted. The first step consisted of a random selection of the classrooms in each school. In the second step, all students in each of the selected classrooms were invited to participate in the study. The number of students in the selected classrooms ranged between 20 and 45. All invited students participated in the study, giving a 100% response rate. The sample size was calculated using methods previously described by Krejcie and Morgan (1970). The overall sample was conceptualized as comprising two subsamples from each type of school (lower secondary school, higher secondary school, and vocational school), with each representing males and females separately, and is summarized in Appendix Table 1. The sample size in this study was calculated with a 5% margin of error, the proportion of the population with a given characteristic was set at 0.5 (to be conservative and provide the maximum sample size), and the Chi-square value for one degree of freedom at 95% confidence interval (3.841). The estimated total sample size was therefore 2,310 students. Nevertheless, because the recruitment was based on number of classrooms rather than individual students, 2,839 participants completed the questionnaire and are included in the study.

### Data Collection, Instruments, and Variables

Participants anonymously completed a Thai-language, self-report questionnaire that collected information related to their socio-demographic and economic background, behaviors, connectedness, parental involvement, and psychological variables. Prior to completing the questionnaire, participants were provided with information regarding the study by trained research assistants. Data collection occurred on school grounds; however, neither teachers nor any staff members affiliated with the school were present during the data collection.

### Measures

#### Primary Outcome: Grit

Grit was assessed using the eight-item Short Grit Scale (Duckworth and Quinn, 2009). The scale consists of items which assess a respondent’s perseverance of effort (e.g., “I am a hard worker” and “Setbacks do not discourage me”). It also included some reverse-scored items that measure consistent pursuit of passionate interest (e.g., “New ideas and projects sometimes distract me from previous ones”) using a five-point Likert scale, ranging from 1 = “Not like me at all” to 5 = “Very much like me.” The scores from all the items are averaged to obtain an overall “Grit score” ranging from 1 (“Not at all Gritty”) to 5 (“Extremely Gritty”). The scale showed a moderate degree of internal consistency in a Thai language version, with Cronbach’s alpha = 0.69. Based on the distribution of scores, the level of Grit among the participants was grouped into three categories: (i) “Low” for scores below the 25^th^ percentile; (ii) “Average” for scores ranging from the 25^th^ to the 75^th^ percentile; and (iii) “High” for scores above the 75^th^ percentile. The full details of the Grit questionnaire and scoring system are reported in Appendix Table 2.

#### Independent Variables

The independent variables which we examine for their relationship with Grit are as follows:

– Connectedness with father (perceived satisfaction of relationship with father); mother (perceived satisfaction of relationship with mother); parents (perceived love and care from parents; perceived mental support from parents); siblings (perceived satisfaction of relationship with siblings); teachers (perceived satisfaction of relationship with teacher); school (perception of school; interest in school subject); and friends (perceived satisfaction of relationship with friends).
– Parental involvement variables were measured with items: “How often did your parents need to ask you to do your homework?”; “How often did your parents notice when you have a problem/feel ill at ease?”; and “How often did your parents tell you when you had done something bad?”
– Socio-economic and demographic variables (e.g., age; gender; education level; parents’ marital status; parents’ level of education; family household income; perceived family financial situation).
– Behavioral variables (e.g., use of alcohol; use of tobacco; use of drugs; engagement in sexual activity) and self-esteem (measured using the Rosenberg Self-Esteem Scale; Rosenberg, 1965). See Appendix Table 3 for more details on this scale and its calculation.

### Data Analysis

The analysis was performed using SPSS 17 (PASW) for Windows (SPSS Inc., Chicago, Illinois, United States). Bivariate analysis was conducted using the *t*-test, ANOVA, or Kruskal–Wallis H test as appropriate. Multiple ordinary least squares linear regression analysis was performed to determine factors associated with Grit. The multivariable model included all independent variables that were significant at *p* < 0.20 in the bivariate analysis. We adjusted for these variables in order to examine each factor for its independent relationship with Grit, removing potential confounding effects. In addition, the following variables were systematically included in the models regardless of their values of *p*: self-esteem; education level; household income; perceived family financial status; mother’s education level; and fathers’ educational level because they are important, potentially modifiable factors. The significance level was set at *p* < 0.05. There was no evidence of multicollinearity.

### Ethics Statement

The study was approved by the Human Experimentation Committee of the Office of Research Ethics of the Research Institute for Health Sciences, Chiang Mai University (Certificate of Ethical Clearance No. 48/2016). Before participating, potential participants were first informed about the study’s objectives, their roles, and their rights to give or not to give any information during the interview, the confidentiality of the personal data, and the way that the findings of the study would be presented. Participants over 18 years of age provided written informed consents prior to participating in the study. For participants under 18 years old, written informed consent was obtained from their legal guardian and written assents to participate in the study were obtained from the participants.

## Results

### Characteristics of the Participants

A total of 2,839 participants completed the questionnaire, including 919 (32.4%) students from lower secondary schools, 1,090 (38.4%) students from higher secondary schools, and 830 (29.2%) from vocational schools. Slightly more than half of the participants were female (53.2%). Most participants were from households with a monthly income between 10,000 and 50,000 Thai Baht (333 and 1,666 USD), and perceived that they earned enough to save and spend (62.0%). The majority reported that their parents were married or lived together (68.0%), but for a substantial proportion (22.4%), the parents were either divorced or separated. For most respondents, the parents’ level of education was at least secondary school: 63.6% for mothers and 62.8% for fathers (Table 1).

**Table 1:** Participants’ general characteristics and bivariate analysis of factors associated with Grit (2,839 participants in total).

Participants Characteristics	Frequency (%)	Mean Grit Score (SD)	^*^ *p*
Grit			
Low	627 (22.1)	N/A	
Average	1,628 (57.3)	N/A	
High	584 (20.6)	N/A	
Sex			0.049
Male	1,330 (46.8)	25.07 (3.47)	
Female	1,509 (53.2)	24.80 (3.78)	
Education			<0.001
Lower secondary school	919 (32.4)	24.97 (3.81)	
Higher secondary school	1,090 (38.4)	25.32 (3.79)	
Vocational school	830 (29.2)	24.34 (3.13)	
Household income			<0.001
< 10,000	319 (11.4)	24.46 (3.26)	
10,000–44,999	1,185 (42.2)	24.94 (3.53)	
≥ 50,000	690 (24.6)	25.52 (4.02)	
Do not know	616 (21.9)	24.50 (3.47)	
Perceived family financial status			<0.001
Enough to save and spend	1740 (62.0)	25.22 (3.63)	
Enough to spend/not save	506 (18.0)	24.42 (3.74)	
Not enough	245 (8.7)	24.16 (3.90)	
Do not know	314 (11.2)	24.68 (3.41)	
Marital status of parents			0.021
Divorced/separated	630 (22.4)	24.60 (3.64)	
Married/live together	1914 (68.0)	25.06 (3.68)	
One/both parents passed died	225 (8.0)	24.56 (3.35)	
Do not know	47 (1.7)	24.96 (3.32)	
Mother’s highest education level			<0.001
Primary education and less	645 (22.9)	24.60 (3.37)	
Secondary/high school	675 (24.0)	24.93 (3.52)	
College/university	1,114 (39.6)	25.28 (3.77)	
Do not know	379 (13.5)	24.40 (3.82)	
Father’s highest education level			<0.001
Primary education and less	562 (20.0)	24.66 (3.36)	
Secondary/high school	629 (22.4)	24.72 (3.49)	
College/university	1,135 (40.4)	25.42 (3.83)	
Do not know	482 (17.2)	24.33 (3.53)	
Ever drank alcohol			<0.001
No	1,530 (54.2)	25.19 (3.73)	
Yes	1,294 (45.8)	24.61 (3.51)	
Ever smoke cigarette			<0.001
No	2,295 (81.2)	25.03 (3.70)	
Yes	530 (18.8)	24.45 (3.31)	
Ever used marijuana			0.280
No	2,607 (92.1)	24.94 (3.63)	
Yes	223 (7.9)	24.67 (3.75)	
Ever bullied and/or assaulted			0.001
No	712 (25.2)	25.34 (3.81)	
Yes	2,118 (74.8)	24.79 (3.57)	
Ever had sex			<0.001
No	2,324 (81.9)	25.03 (3.71)	
Yes	515 (18.1)	24.92 (3.64)	
Self-esteem^**^			<0.001
Low (<15)	592 (21.5)	23.18 (3.43)	
Normal (15–25)	2,117 (76.8)	25.27 (3.42)	
High (>25)	49 (1.8)	30.06 (4.95)	

* *Value of p measures statistical significance of differences in Mean Grit score between participant groups using Student’s-t test or analysis of variance (ANOVA) as appropriate*.

** *Measured using Rosenberg self-esteem scale as described in* Appendix Table 3.

The majority felt that they were well connected to their parents and friends. For example, satisfaction with the relationship with mother, father, and friends was 88.9%, 78.6%, and 91.5%, respectively. Similarly, most felt that their parents (80.8%) and friends (70.4%) gave them mental/emotional support. The proportion of those who reported parental support, and involvement in the positive ways was generally high. However, many reported that their parents needed to ask them to do homework (92.3%) and chores around the house (94.2%) or told them when they had done something bad (97.8%).

There were also high levels of connectedness with the school and teacher. Most participants were satisfied with the relationship with their teacher (74.6%), liked the school (84.1%), were interested in the subject at school (89.1%), and felt that their performance at school was at least average (82.9%). In terms of substance use, 45.8% reported ever drinking alcohol, 18.8% ever smoked a cigarette, and 7.9% ever used marijuana. Around 18.1% reported having had sex and 21.5% were classified as having low self-esteem (Table 2).

**Table 2:** Participants’ perception and experience related to connectedness and bivariate analysis of factors associated with Grit (2,839 participants in total).

Participants Characteristics	Frequency (%)	Mean Grit Score (SD)	^*^ *p*
Perceived satisfaction of relation with mother			<0.001
Not satisfied/not sure	313 (11.1)	23.98 (4.09)	
Satisfied	2,502 (88.9)	25.05 (3.56)	
Perceived satisfaction with relationship with father			<0.001
Not satisfied/not sure	600 (21.4)	24.34 (3.94)	
Satisfied	2,209 (78.6)	25.12 (3.59)	
You feel love and care from your parents			<0.001
Other	404 (14.3)	23.80 (3.76)	
All the time/quite so much	2,413 (85.7)	25.12 (3.59)	
You feel that your parents give you mental support			<0.001
Other	541 (19.2)	23.92 (3.54)	
All the time/quite so much	2,273 (80.8)	25.17 (3.63)	
Perceived satisfaction with relationship with friend			<0.001
Not satisfied/not sure	239 (8.5)	24.14 (4.06)	
Satisfied	2,582 (91.5)	25.00 (3.59)	
You feel that your friends are take care/ kind to you			<0.001
Other	663 (23.5)	24.30 (3.59)	
Always/a lot	2,161 (76.5)	25.12 (3.64)	
You feel that your friends give you mental support			<0.001
No	833 (29.6)	24.43 (3.66)	
Yes	1984 (70.4)	25.13 (3.61)	
Perceived satisfaction with relationship with teacher			<0.001
Other	716 (25.4)	24.12 (3.66)	
Satisfied	2,104 (74.6)	25.19 (3.60)	
Perception of one’s school			<0.001
Other	448 (15.9)	24.02 (3.59)	
I like my school	2,371 (84.1)	25.09 (3.62)	
Interest in subjects at school			<0.001
Other	308 (10.9)	23.71 (3.69)	
Interested	2,525 (89.1)	25.07 (3.61)	
Perceived performance at school			<0.001
Low (lower middle/low)	482 (17.1)	23.89 (3.17)	
Middle	1,432 (50.7)	24.74 (3.46)	
Top (top/upper middle)	910 (32.2)	25.78 (3.98)	
How often did your parents need to ask you to do your homework?			< 0.001
Never	208 (7.4)	26.18 (4.29)	
Ever (sometime/regularly/once in a while)	2,602 (92.3)	24.83 (3.57)	
Do not know	9 (0.3)	27.44 (3.94)	
How often did your parents make you do chores around the house?			0.001
Never	149 (5.3)	25.93 (4.61)	
Ever (sometime/regularly/occasionally)	2,652 (94.2)	24.87 (3.57)	
Do not know	14 (0.5)	26.64 (3.85)	
How often your parents notice when you have a problem/ feel ill at ease?			0.054
Never	324 (11.5)	24.48 (4.05)	
Ever (sometime/regularly/occasionally)	2,376 (84.6)	25.00 (3.54)	
Do not know	109 (3.9)	24.80 (4.37)	
How often did your parents tell you when you had done something bad?			0.089
Never	51 (1.8)	25.84 (4.30)	
Ever (sometime/regularly/occasionally)	2,750 (97.8)	24.92 (3.63)	
Do not know	12 (0.4)	23.58 (2.67)	
How often did your parents let you know when you had done a good job?			0.004
Never	147 (5.2)	24.01	
Ever (sometime/regularly/occasionally)	2,576 (91.6)	24.97	
Do not know	90 (3.2)	25.43	
How many times have you argued or had a fight with at least one of your parents?			0.005
Never	457 (16.3)	25.42 (4.05)	
Ever (sometime/regularly/occasionally)	2,302 (82.0)	24.82 (3.56)	
Do not know	47 (1.7)	25.21 (3.24)	

* *Value of p measures statistical significance of differences in Mean Grit score between participant groups using Student’s-t test or analysis of variance (ANOVA) as appropriate*.

### Factors Associated With Grit

Many factors were significantly related to Grit in the bivariate analysis (Tables 1 and 2). In the adjusted model (Table 3), Grit scores were higher among participants who perceived that their parents gave them mental/emotional support (*β* = 0.49; *p* = 0.028), those who were satisfied with their relationship with their teachers (*β* = 0.44; *p* = 0.008), and those who were interested in the subjects at school (*β* = 0.61; *p* = 0.01). Two variables of parental involvement were related to Grit. Participants who responded that their parents who had to ask them to do homework (*β* = −0.69; *p* = 0.012) and had ever told them if they have done something bad (*β* = −1.09; *p* = 0.02) had lower Grit scores compared to those who responded never.

**Table 3:** Multivariable analysis of factors associated with Grit.

	*n*	*β* ^*^	95% CI	*p*
Self-esteem				
Low (<15)	592	Ref		
Normal (15–25)	2,117	1.287	0.959, 1.615	<0.001
High (>25)	49	5.266	4.242, 6.290	<0.001
Sex				
Male	1,330	Ref		
Female	1,509	−0.384	−0.664, −0.088	0.006
Educational level				
Lower secondary school	919	Ref		
Higher secondary school	1,090	0.304	−0.036, 0.599	0.072
Vocational school	830	−0.459	−0.870, −0.047	0.029
Perceived family financial status				
Lower secondary school	1740	Ref		
Higher secondary school	506	−0.418	−0.780, −0.056	0.024
Vocational school	245	−0.619	−1.119, −0.119	0.015
Lower secondary school	314	−0.082	−0.527, 0.363	0.717
You feel that your parents give you mental support				
Other	541	Ref		
All the time/quite so much	2,273	0.499	0.053, 0.946	0.028
Perceived satisfaction of relation with teacher				
Other	716	Ref		
Satisfied	2,104	0.437	0.113, 0.761	0.008
Interest in the subject at school				
Other	308	Ref		
Interested	2,525	0.612	0.147, 1.077	0.010
Perceived performance at school				
Low (lower middle/low)	482	Ref		
Middle	1,432	1.024	0.612, 1.436	<0.001
Top (top/upper middle)	910	0.436	0.056, 0.815	0.024
How often did your parents need to ask you to do your homework?				
Never	208	Ref		
Ever (sometime/regularly/occasionally)	2,602	−0.686	−1.223, −0.148	0.012
Do not know	9	3.077	0.533, 5.621	0.018
How often did your parents tell you when you had done something bad?				
Never	51	Ref		
Ever (sometime/regularly/occasionally)	2,750	−1.088	−2.022, −0.154	0.022
Do not know	12	−4.609	−7.013, −2.205	<0.001

* *Multivariate associations were adjusted for all covariates included in the table, although this table includes only variables that showed significant differences (p = 0.05 or less)*.

In addition, we found that Grit scores were on average higher among participants with normal self-esteem (*β* = 1.29; *p* < 0.001) and high self-esteem (*β* = 5.27; *p* < 0.001) compared to those with low self-esteem. Female participants had lower Grit scores compared to male participants (*β* = −0.38; *p* = 0.006). Although there was no statistical difference between the participants in terms of household income, those who perceived that they did not earn enough to save (*β* = −0.418; *p* = 0.024), or just did not earn enough (*β* = −0.619; *p* = 0.015), had lower Grit scores than those who perceived earning enough to save. Grit scores were also higher among the participants who were classified as average (*β* = 1.02; *p* < 0.001) and top (*β* = 0.44; *p* = 0.024) in terms of their performance at school. However, the Grit scores of participants from vocational schools were lower compared to those from secondary school (*β* = −0.46; *p* = 0.029).

## Discussion

To the best of our knowledge, this study is the first to assess the association of connectedness and parental involvement, in both positive and negative ways, with Grit among adolescents in Thailand. Existing studies have unlikely to examine positive and/or negative parental involvement in the detail reported here. This provides new insights into the social factors that influence Grit. Strength of this study is that it is based on a large sample of Thai adolescents, randomly recruited from all public and private secondary schools, high schools, and vocational schools in urban Chiang Mai. The data thus allow for generalizability of our results for Chiang Mai city students. We found that being satisfied with relationships with the teacher, perceived parental support, interest in subjects at school, and positive parental involvement were predictors of Grit.

There is evidence in the United States showing a positive correlation between Grit and school motivation, perceived peer support, perceived teacher support, and perceived school safety (Eskreis-Winkler et al., 2014). Students’ experiences in school including school engagement, relationships with adults and peers, school culture, and self-reported GPA were found to be most strongly related to Grit (Wanzer, 2018). Similarly, in our study, we found a positive relationship between Grit and connectedness to school; specifically, in those reporting a satisfactory relationship with teacher and interest in school subjects. In our study, there was a positive correlation between perceived parents’ provision of mental support with Grit. However, satisfaction with relationship with fathers and mothers and perceived love and care from parents, which were statistically significant in bivariate analysis, lost significance in the multivariable model. It is possible that adolescents who receive positive mental support from parents perceived this to be manifest as love and care.

In this study, we found participants who responded that their parents who had to ask them to do homework and had ever told them if they have done something bad had lower Grit scores compared to those who responded that this had never happened. It can be explained that participants who had self-discipline had no need from parents to ask them to do their own homework. In case of telling children when they do something bad in Thai context, particularly among laypeople in which parents and children normally talk to each other a lot. Parents will tell their children straightforwardly and sometimes emotionally in loud voices if they do something wrong. Many children tend to interpret this as parents blaming them. So, for those who had never been told by parents that they did something bad can indicate that they had no need to be told/blamed by parents because of their good behavior or self-discipline. It is likely that students who perceived parental involvement to be critical had lower Grit, reinforcing research showing that Grit is built on positive connections that build self-esteem.

Indeed, the present study showed that Grit scores were on average higher among participants with normal and high self-esteem. Self-esteem can also predict perseverance and consistent interest, which are components of Grit (Weisskirch, 2018). One study demonstrated that self-esteem mediated the relationship between Grit and life satisfaction (Li et al., 2018). Thus, it is reasonable to conclude that higher self-esteem would associate with higher Grit because individuals with a positive self-evaluation may persevere more in the face of challenges.

This study also revealed that adolescents who perceived that they belonged to households with insufficient income had lower Grit scores than those whose household income was sufficient and allowed for savings. Although there is a paucity of research on the relationship between subjective socioeconomic status (SES) and Grit, studies on students’ persistence in higher education consistently found that students from a low socioeconomic background were less likely to persist through college (Pascarella and Terenzini, 1991, 2005; Reason, 2009).

The evidence thus far is inconclusive on gender differences in Grit levels. Our study, like others (Christensen and Knezek, 2014; Tiittanen, 2014) found that male students had higher Grit than female students. In a patriarchal society like Thailand, men’s traditional roles as the leader and main provider for the family may contribute to men’s perseverance and Grit in the pursuit of long-term goals. However, other studies, some in patriarchal culture, reported only marginal or no significant differences in Grit level between males and females (Ali and Rahaman, 2012; Flaming and Granato, 2017). Research also indicates that differences between genders vary. For example, Duckworth and Gross (2014) found that girls had more self-control than boys. Given that self-control and Grit were strongly correlated, though not perfectly (Duckworth and Gross, 2014), this would suggest that females with higher self-control have higher Grit. However, it was not clear whether gender differences in self-control and/or self-discipline persisted into adulthood (Duckworth and Seligman, 2006).

## Limitations

This study has some limitations. First, no causal inference can be drawn from the documented associations due to the cross-sectional study design. Future, longitudinal studies would be of particular value in assessing the causative factors for Grit. Second, although participants were recruited from all 21 schools in urban Chiang Mai, it is unclear to what extent the results can be generalized to other settings in Thailand, given the significant local and regional socioeconomic and demographic heterogeneity in the country. Some of the actual differences of Grit between categories were small and might not be of public health significance. These might occur because of large sample size. Readers may need to accept the results with caution. Finally, we acknowledge that our results depend on self-report from participants which may induce some bias.

## Conclusion and Implications

This brief research report provides evidence of factors associated with Grit in Thai students. The data showed that connectedness to social agents (parents, peers, and teachers) and positive parental involvement had positive correlation to Grit in Thai students in Chiang Mai. However, our findings are preliminary, and further research is required to understand the relationships between connectedness, positive parental involvement and Grit as well as their impacts on health, academic, and life success. Thailand is interested in improving the standard of its educational programs, so that it may catch up with its South-East Asian neighbors such as Vietnam and other neighboring countries. A deeper understanding of the factors that build Grit and therefore academic outcomes would contribute to this project. This study has identified potential home and school-based factors for further study and potentially for interventions, for urban Thai students in Chiang Mai and across the country.

## Data Availability Statement

The original contributions presented in the study are included in the article/Supplementary Material, further inquiries can be directed to the corresponding author.

## Ethics Statement

The studies involving human participants were reviewed and approved by the Human Experimentation Committee of the Office of Research Ethics of the Research Institute for Health Sciences, Chiang Mai University. Written informed consent to participate in this study was provided by the participants’ legal guardian/next of kin.

## Author Contributions

AT and KT conceived the study. AT, KT, PM, and KS collected the data. AT, MK, and CB contributed to data analysis and interpretation. AT drafted the paper. All authors contributed to the article and approved the submitted version.

## Funding

This study was supported by internal Chiang Mai University Research Funds. The funder played no role in study design or interpretation.

## Conflict of Interest

The authors declare that the research was conducted in the absence of any commercial or financial relationships that could be construed as a potential conflict of interest.

## Publisher’s Note

All claims expressed in this article are solely those of the authors and do not necessarily represent those of their affiliated organizations, or those of the publisher, the editors and the reviewers. Any product that may be evaluated in this article, or claim that may be made by its manufacturer, is not guaranteed or endorsed by the publisher.
