# Supplementary material for: The Association Between Connectedness and Grit Among Thai In-school Adolescents in Urban Chiang Mai, Thailand
Source: Front Psychol. 2022 Mar 28;13:809508. doi: 10.3389/fpsyg.2022.809508 (PMC8997333; doi:10.3389/fpsyg.2022.809508)
Supplement: Supplementary file 1 [file Data_Sheet_1.docx]

**Appendix Tables**

**APPENDIX TABLE 1:** Sampling procedure by schooling level and sex

| **School level and sex groupings** | **Population size** | **Required sample size** |
| --- | --- | --- |
| Lower secondary school (age 13-14) males | 2050 | 385 |
| Lower secondary school (age 13-14) females | 2180 | 389 |
| Higher secondary school (age 16-17) males | 1696 | 372 |
| Higher secondary school (age 16-17) females | 2283 | 391 |
| Vocational school (age 16-17) males | 2810 | 403 |
| Vocational school (age 16-17) females | 1639 | 370 |
| **Total sample size** |  | 2310 |

**APPENDIX TABLE 2:** Short Grit Scale and scoring system

| Please respond to the following 8 items.  Be honest – there are no right or wrong answers. | Very much like me | Mostly like me | Somewhat like me | Not much like me | Not like me at all |
| --- | --- | --- | --- | --- | --- |
| 1. New ideas and projects sometimes distract me from previous ones. * |  |  |  |  |  |
| 2 Setbacks don’t discourage me. |  |  |  |  |  |
| 3. I have been obsessed with a certain idea or project for a short time but later lost interest. * |  |  |  |  |  |
| 4. I am a hard worker. |  |  |  |  |  |
| 5. I often set a goal but later choose to pursue a different one. * |  |  |  |  |  |
| 6. I have difficulty maintaining my focus on projects that take more than a few months to complete. * |  |  |  |  |  |
| 7. I finish whatever I begin. |  |  |  |  |  |
| 8. I am diligent. |  |  |  |  |  |

Scoring:

1. For questions 2, 4, 7 and 8 assign the following points:

5 = Very much like me

4 = Mostly like me

3 = Somewhat like me

2 = Not much like me

1 = Not like me at all

2. For questions 1, 3, 5 and 6 assign the following points:

1 = Very much like me

2 = Mostly like me

3 = Somewhat like me

4 = Not much like me

5 = Not like me at all

Add up all the points and divide by 8. The maximum score on this scale is 5 (extremely gritty), and the lowest score on this scale is 1 (not at all gritty).

**APPENDIX TABLE 3**: Rosenberg self-esteem scale

Please tell us about how you feel

| STATEMENT | Strongly Agree | Agree | Disagree | Strongly Disagree |
| --- | --- | --- | --- | --- |
| 1. On the whole, I am satisfied with myself. |  |  |  |  |
| 2. At times I think I am no good at all. |  |  |  |  |
| 3. I feel that I have a number of good qualities |  |  |  |  |
| 4. I am able to do things as well as most other people. |  |  |  |  |
| 5. I feel I do not have much to be proud of. |  |  |  |  |
| 6. I certainly feel useless at times. |  |  |  |  |
| 7. I feel that I'm a person of worth. |  |  |  |  |
| 8. I wish I could have more respect for myself. |  |  |  |  |
| 9. All in all, I am inclined to think that I am a failure. |  |  |  |  |
| 10. I take a positive attitude toward myself. |  |  |  |  |

**Rosenberg Self-Esteem Scale (RSE)**

**Author:** Morris Rosenberg

The purpose of the 10 item RSE scale is to measure self-esteem. Originally the measure was designed to measure the self-esteem of high school students. However, since its development, the scale has been used with a variety of groups including adults, with norms available for many of those groups.

**Scoring:** As the RSE is a Guttman scale, scoring can be a little complicated. Scoring involves a method of combined ratings. Low self-esteem responses are “disagree” or “strongly disagree” on items 1, 3, 4, 7, 10, and “strongly agree” or “agree” on items 2, 5, 6, 8, 9. Two or three out of three correct responses to items 3, 7, and 9 are scored as one item. One or two out of two correct responses for items 4 and 5 are considered as a single item; items 1, 8, and 10 are scored as individual items; and combined correct responses (one or two out of two) to items 2 and 6 are considered to be a single item.

The scale can also be scored by totalling the individual 4-point items after reverse-scoring the negatively worded items.

**Reliability:** The RSE demonstrates a Guttman scale coefficient of reproducibility of .92, indicating excellent internal consistency. Test-retest reliability over a period of 2 weeks reveals correlations of .85 and .88, indicating excellent stability.

**Validity:** Demonstrates concurrent, predictive and construct validity using known groups. The RSE correlates significantly with other measures of self-esteem, including the Coopersmith Self-Esteem Inventory. In addition, the RSE correlates in the predicted direction with measures of depression and anxiety.

**Reference:** Rosenberg, M. (1979). Conceiving the Self. New York: Basic Books.
